# Comprehensive Preoperative Strategy and Multidisciplinary Collaboration for Safe Completion Pneumonectomy: A Case Report

**DOI:** 10.7759/cureus.103640

**Published:** 2026-02-15

**Authors:** Tomonari Oki, Shuhei Iizuka, Yoshifumi Kunii, Tokimitsu Hibino, Toru Nakamura

**Affiliations:** 1 Department of General Thoracic Surgery, Seirei Hamamatsu General Hospital, Hamamatsu, JPN; 2 Department of Cardiovascular Surgery, Seirei Hamamatsu General Hospital, Hamamatsu, JPN; 3 Department of Anesthesiology, Seirei Hamamatsu General Hospital, Hamamatsu, JPN

**Keywords:** cardiopulmonary bypass, completion pneumonectomy, intrapericardial control, multidisciplinary collaboration, superior vena cava

## Abstract

Completion pneumonectomy (CP) is a technically demanding procedure associated with significant morbidity and mortality, primarily due to dense hilar adhesions and anatomical distortions from previous surgeries. Right CP is particularly hazardous given potential intrapericardial involvement. Comprehensive preoperative planning and multidisciplinary collaboration are essential to mitigate the risk of catastrophic intraoperative hemorrhage. A 57-year-old woman with a history of right middle lobectomy for lung cancer nine years prior presented with a local recurrence necessitating a right CP. Given the anticipation of intrapericardial adhesions and the potential need for cardiopulmonary bypass (CPB), a meticulous preoperative strategy was devised. The patient was placed in a modified left lateral decubitus position with a 45° posterior pelvic rotation, allowing simultaneous access for a posterolateral thoracotomy and femoral CPB cannulation. Strategic central venous access was established to ensure adequate venous return in the event of superior vena cava (SVC) clamping. Intraoperatively, dense adhesions involving the extrapericardial SVC were identified. In contrast, the pericardial space was relatively free of adhesions, allowing for the safe intrapericardial control of the pulmonary vessels. By establishing proximal control within the pericardium and distal control at the brachiocephalic veins, the SVC was safely managed. The procedure was completed without the need for CPB initiation. The postoperative course was uneventful, and the patient remained recurrence-free at the one-year follow-up. Successful CP requires more than surgical technicality; it demands a robust multidisciplinary strategy. Meticulous preparation regarding standby CPB, optimized patient positioning, and proactive vascular control are critical components in ensuring safety and achieving favorable outcomes in high-risk thoracic procedures.

## Introduction

Lobectomy became the gold standard for non-small cell lung cancer (NSCLC) in the 1960s, providing survival outcomes equivalent to pneumonectomy with superior quality-of-life preservation [1]. Nevertheless, the risk of local recurrence or secondary lung cancer persists, potentially requiring completion pneumonectomy (CP) [2]. CP is among the most technically demanding and high-risk procedures in thoracic surgery [3,4]. Intraoperative mortality, postoperative mortality, and postoperative complications have been reported to range from 0-2.3%, 7.4-14.9%, and up to 53%, respectively [5,6]. Right CP significantly reduces the pulmonary vascular bed, causing increased pulmonary artery systolic pressure and acute right ventricular (RV) dilatation, frequently leading to postoperative right heart dysfunction. This condition, often marked by increased RV end-diastolic volume and reduced ejection fraction, is more pronounced in right-sided procedures due to greater loss of pulmonary vasculature, particularly in elderly patients with low preoperative partial arterial oxygen [7]. Moreover, the anatomical requirement for the right main pulmonary artery to pass behind the ascending aorta and superior vena cava (SVC) complicates surgical access compared to the left side, leading to a higher degree of difficulty in right CP [8]. Despite these risks, it remains an essential treatment modality for patients with recurrent or secondary lung cancer following prior ipsilateral anatomical pulmonary resection [4]. Dense hilar adhesions and potential intrapericardial involvement due to prior surgery significantly increase the risk of life-threatening intraoperative hemorrhage [3]. Although intrapericardial control of pulmonary vessels is frequently required [4], intrapericardial adhesions may render such dissection hazardous. In this context, cardiopulmonary bypass (CPB), which can be a valuable adjunct in the surgical treatment of selected tumors and vascular malformations involving large or central pulmonary vessels [9], serves as a potential safety measure [10]. Although right CP is an exceptionally high-risk procedure [3], there is a paucity of detailed reports addressing comprehensive preoperative strategies and intraoperative management. Herein, we present a case of successful right CP performed with meticulous preoperative planning and multidisciplinary collaboration.

## Case presentation

**Figure 1:**
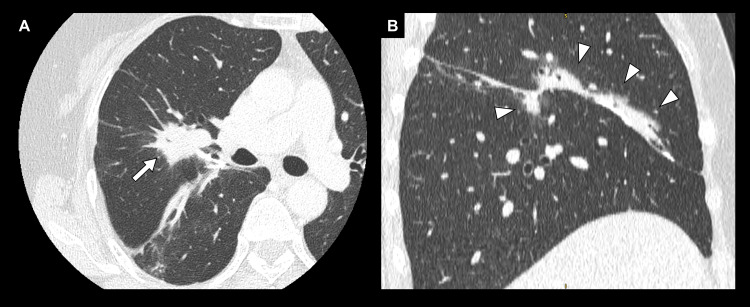
Computed tomography findings at recurrence (A) Axial view showing a 26 mm nodule (white arrow) involving the previous stapler line of the upper lobe. (B) Sagittal view showing a 32 mm mass along the stapler line (white arrowheads).

A 57-year-old woman, a non-smoker with a history of depression, was referred to our hospital for a mass of the right lung incidentally detected on a computed tomography scan performed to evaluate chest pain (Figure 1). The chest pain resolved spontaneously and was found to be unrelated to the mass.

**Figure 2:**
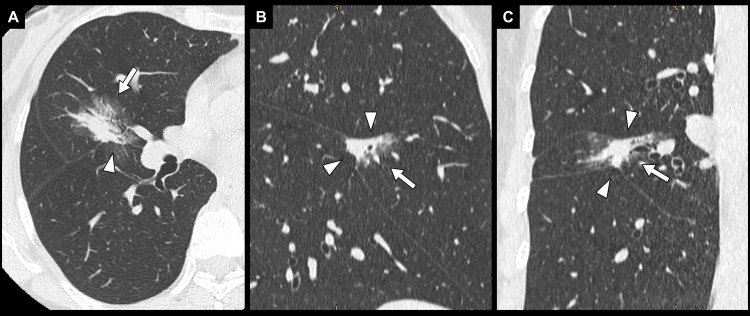
Computed tomography of the right middle lobe lung cancer nine years prior A 25 mm part-solid ground-glass nodule with a 15 mm consolidation component (white arrows) is located in the right middle lobe, adjacent to both the upper and lower lobes (white arrowheads). (A) Axial view. (B) Sagittal view. (C) Coronal view.

She had a history of right middle lobe lung cancer nine years prior. The previous lung cancer had been a part-solid ground-glass nodule (GGN) with a maximum diameter of 25 mm and a solid component of 15 mm adjacent to the upper and lower lobes (Figure 2), for which she underwent a right middle lobectomy with mediastinal lymph node dissection.

**Figure 3:**
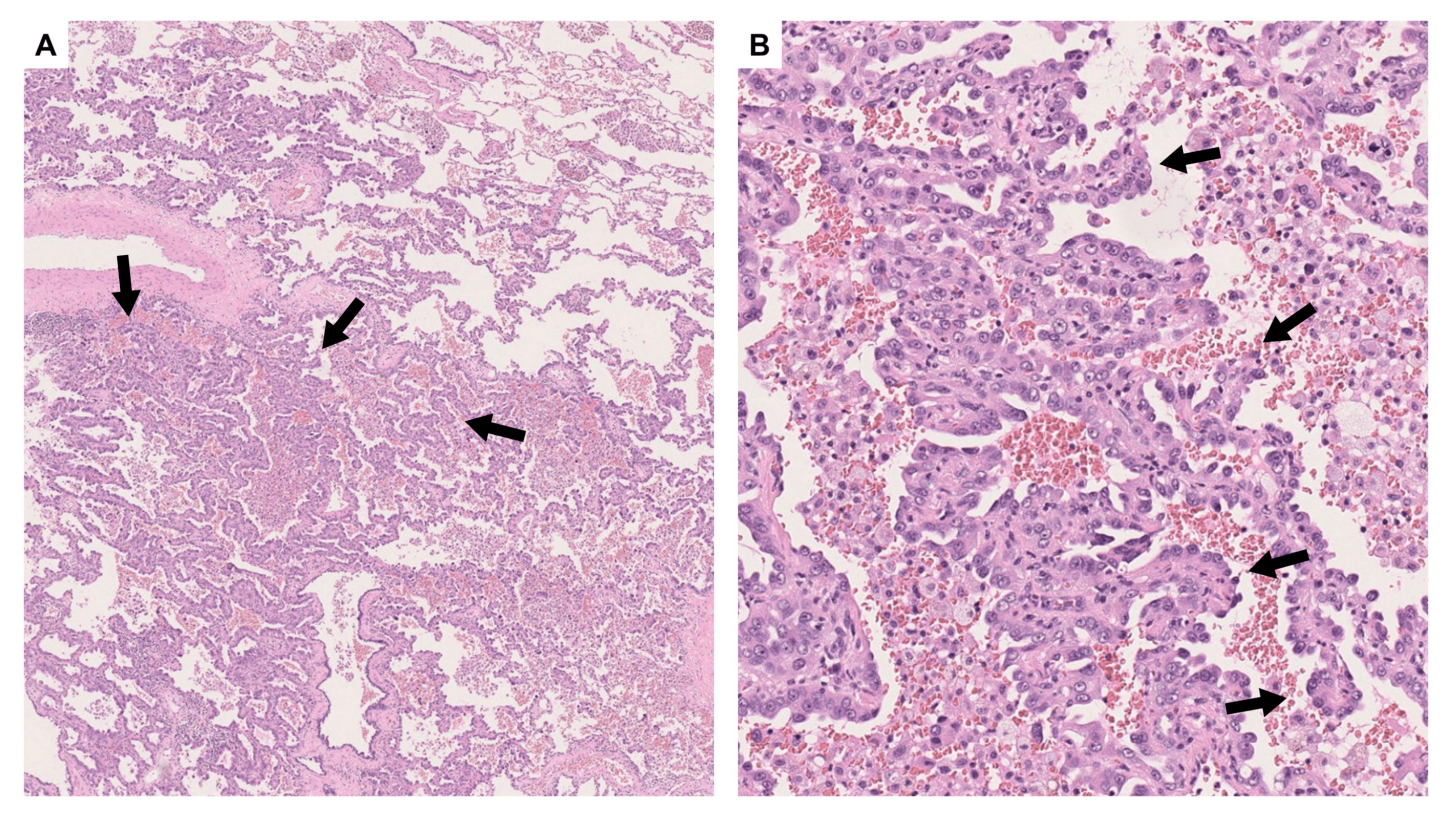
Pathological findings of the right middle lobe lung cancer nine years prior The tumor cells showed a papillary growth pattern (black arrows). (A) Hematoxylin and eosin stain, ×1.25. (B) Hematoxylin and eosin stain, ×10.

Pathological examination demonstrated a 23 mm papillary adenocarcinoma with a 12 mm invasive component, without lymphovascular or pleural invasion, classified as pT1bN0M0, stage IA2 (Figure 3). No alterations in the epidermal growth factor receptor or anaplastic lymphoma kinase genes were identified. All resection margins were confirmed to be negative for malignancy. The pathological findings confirmed the appropriateness of right middle lobectomy as the definitive procedure [11]. The patient remained disease-free for five years and was discharged from follow-up.

**Figure 4:**
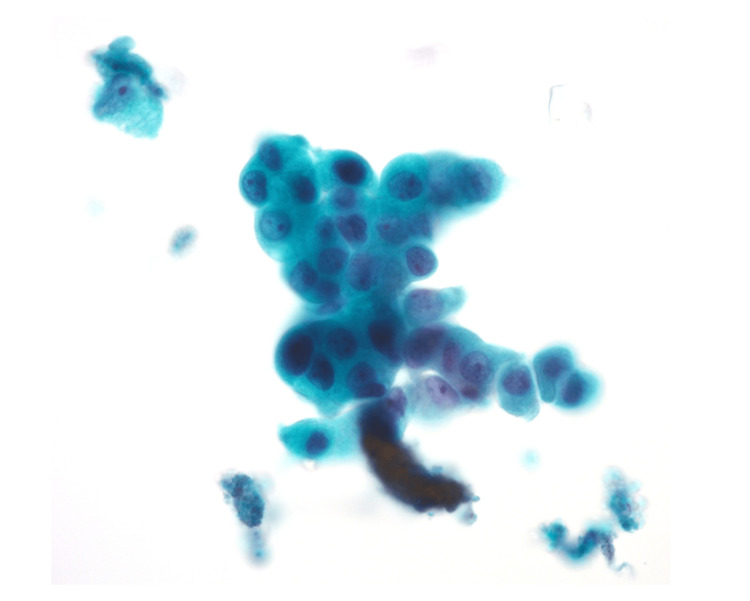
Bronchoscopic cytology findings Malignant cells with enlarged ovoid nuclei, conspicuous nucleoli, and finely granular chromatin (Papanicolaou stain, ×40).

The newly emerged mass arose along the stapler line of the previous surgery (Figure 1), and its close proximity to the prior lesion (Figure 2) favored local recurrence over a second primary lung cancer. Bronchoscopic cytology revealed adenocarcinoma (Figure 4); however, biomarker analysis, including genetic alterations and programmed death-ligand 1 expression, could not be performed because of insufficient tissue. On bronchoscopy, there was no evidence of extraluminal compression, with a normal appearance of the bronchial mucosa and the previous right middle lobe bronchial stump. Whole-body positron emission tomography and brain magnetic resonance imaging showed no distant metastasis.

Treatment planning

Because the tumor involved the staple lines of both the residual upper and lower lobes, a right CP was required for complete resection. Given the substantial morbidity associated with this procedure, alternative treatment options were carefully evaluated in a multidisciplinary tumor board discussion comprised of thoracic surgeons, pulmonologists, medical oncologists, and radiologists. However, optimal systemic therapy could not be determined due to the lack of biomarker data. Re-biopsy was deemed unfeasible because no bronchus directly accessed the tumor. Radiotherapy was considered inappropriate, as it would have required a broad irradiation field and accurate tumor delineation was hindered by overlap with the high‑attenuation area along the stapler line. Conversely, the patient was relatively young (57 years) without significant comorbidities. Her Eastern Cooperative Oncology Group Performance Status (ECOG PS) was 0, as she had no restrictions on her activities of daily living or physical exertion. Pulmonary function testing (Table 1) demonstrated preserved respiratory reserve (forced vital capacity (FVC): 2.9 L (116.9%); forced expiratory volume in one second (FEV1): 2.27 L (110.2%)). The predicted postoperative FEV1 after right CP was 1.2 L (58.3%). Given the absence of emphysema or interstitial lung disease, diffusing capacity of the lung for carbon monoxide assessment was not performed.

**Table 1:** Results of preoperative pulmonary function testing prior to completion pneumonectomy FVC: forced vital capacity; FEV1: forced expiratory volume in one second; FEV1/FVC: forced expiratory volume in one second-to-forced vital capacity ratio

Parameter	Measured value	Predicted value	% of predicted
FVC (L)	2.90	2.48	116.9%
FEV1 (L)	2.27	2.06	110.2%
FEV1/FVC ratio (%)	78.3	77.2	101.4%

**Figure 5:**
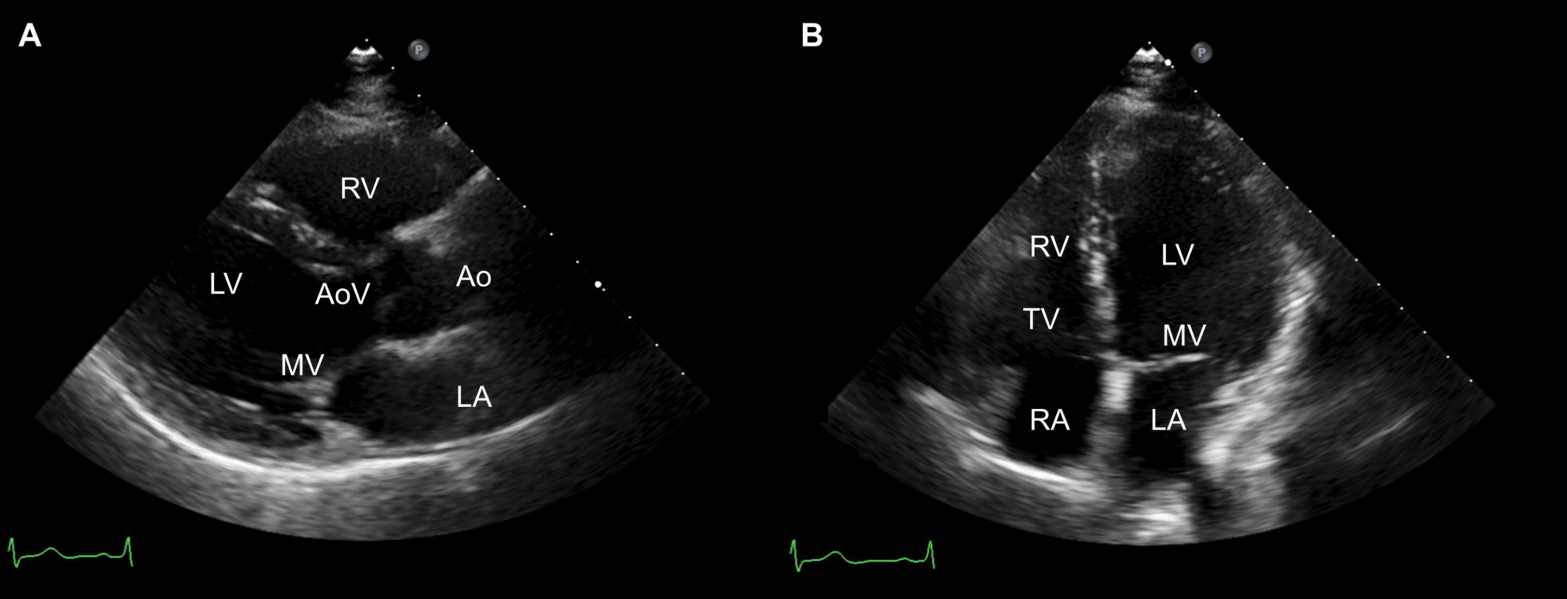
Preoperative echocardiography prior to completion pneumonectomy Transthoracic echocardiography demonstrated normal left ventricular function (ejection fraction, 66.6%) with no evidence of right ventricular enlargement. (A) Parasternal long‑axis view. (B) Apical four‑chamber view. Ao: aorta; AoV: aortic valve; LA: left atrium; LV: left ventricle; MV: mitral valve; RA: right atrium; RV: right ventricle; TV: tricuspid valve

Echocardiography revealed normal left ventricular function (ejection fraction: 66.6%) and no RV enlargement (Figure 5). A stair-climbing test demonstrated that the patient could climb more than five flights of stairs, suggesting she could tolerate the procedure [12,13]. Consequently, pulmonary perfusion scintigraphy was omitted. Because of significant surgery-related anxiety associated with the patient's depression, a psychiatric consultation was obtained to optimize anxiolytic therapy. The patient was managed with paroxetine (10 mg/day), ethyl loflazepate (2 mg/day), lorazepam (1.5 mg/day), and lemborexant (5 mg/day) for a body weight of 64 kg. Additionally, repeated clinical counseling was provided. Following these interventions and comprehensive discussions, the patient ultimately consented to proceed with a right CP.

Surgical preparation

A major concern in assessing the feasibility of CP was the extent of mediastinal lymph node dissection performed during the prior surgery. Review of the operative records indicated that the right middle lobectomy was performed through an 8 cm posterolateral thoracotomy and lymphadenectomy had extended to the proximal main pulmonary artery (PA) after the division of the azygos vein. Specifically, dense adhesions between the intrapericardial SVC and the right main PA were anticipated, and injury in this region could result in catastrophic hemorrhage. Given this risk, a comprehensive preoperative plan was deemed necessary. To ensure optimal surgical preparation, a multidisciplinary team including thoracic and cardiovascular surgeons, anesthesiologists, perfusionists, and nurses conducted a comprehensive review and shared essential information regarding the intraoperative decision-making process. The surgical team planned to institute CPB if intrapericardial adhesions precluded safe dissection, while standby CPB was prepared for immediate initiation even in the absence of such adhesions. Regarding the surgical approach, ensuring an optimal view of the proximal right main PA was crucial. Supine approaches, including a hemiclamshell incision or median sternotomy, provide excellent access to the right main PA and the intrapericardial space and facilitate CPB cannulation. However, these approaches are less suitable for extensive adhesiolysis in the posterior thoracic cavity. In this case, since subcarinal lymph node dissection had been performed via a posterior approach during the initial surgery, dense adhesions were anticipated at the posterior hilum. Therefore, we opted for a posterolateral incision in the lateral decubitus position, extended anteriorly as needed, which allowed comprehensive thoracic adhesiolysis while still providing sufficient access to the proximal right main PA. Although the ascending aorta and right atrium were accessible via the right thoracic cavity, potential interference of the CPB circuit with the surgical field was a concern; therefore, femoral artery and vein cannulation were selected.

The criteria for initiating CPB were established as follows. In cases where extensive intrapericardial adhesions made it difficult to identify the right main PA or pulmonary veins, CPB would be initiated prior to the commencement of dissection. If the anatomy could be identified but adhesions were present around the right main PA, the femoral artery and vein would be exposed before intrapericardial dissection to allow for emergency cannulation and rapid CPB initiation in the event of hemorrhage. In the absence of any intrapericardial adhesions, pre-exposure of the femoral vessels would not be performed.

An epidural catheter was placed one day before surgery, considering the need for systemic anticoagulation during CPB. An electrosurgery unit (ESU) and an ultrasonic surgical device (USD) were allocated for the thoracic procedure, with a separate ESU designated for the femoral procedure. A thoracoscope was prepared for intraoperative visualization and documentation.

**Figure 6:**
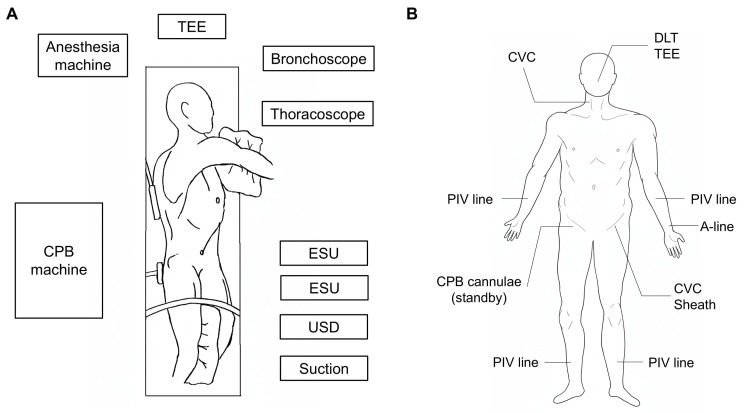
Operating room layout (A) Diagram of the operating room setup and patient positioning. TEE is positioned at the patient's head. The anesthesia machine and CPB machine are placed on the patient's right (dorsal) side. Monitors for bronchoscopy and thoracoscopy, ESUs, a USD, and suction systems are positioned on the left (ventral) side. The patient is placed in a modified left lateral decubitus position with a 45° posterior pelvic rotation, which allowed a simultaneous posterolateral thoracotomy and access to the femoral vessels. (B) Lines and monitoring devices placed at anesthesia induction. A DLT and TEE probe are inserted orally. A CVC is inserted via the right internal jugular vein. Another CVC and sheath are inserted via the left femoral vein. PIV lines are secured in the extremities. An A-line is inserted via the left radial artery. The right femoral artery and vein are prepared for potential CPB cannulae insertion. TEE: transesophageal echocardiography; CPB: cardiopulmonary bypass; ESUs: electrosurgery units; USD: ultrasonic surgical device; DLT: double-lumen endotracheal tube; CVC: central venous catheter; PIV: peripheral intravenous; A-line: arterial line The illustrations were generated using Microsoft Paint, version 11, and Microsoft PowerPoint 2021 (Microsoft Corporation, Redmond, Washington, United States).

The operating room was configured to maximize procedural efficiency. Transesophageal echocardiography (TEE) was positioned at the head of the patient. The anesthesia machine and CPB system were placed on the right (dorsal) side, whereas bronchoscopic and thoracoscopic monitors, ESUs, the USD, and the suction system were arranged on the left (ventral) side (Figure 6A).

The patient was intubated with a 35-Fr left-sided double-lumen endobronchial tube. Prior superior mediastinal lymph node dissection raised concern for dense adhesions involving the extrapericardial SVC and the potential need for SVC clamping. Accordingly, central venous access was established via the right internal jugular and the left femoral veins to ensure adequate venous return. A large-bore sheath for rapid infusion was additionally placed in the left femoral vein, whereas the right femoral vein was preserved for CPB cannulation (Figure 6B). TEE was employed for continuous cardiac monitoring and to facilitate prompt CPB initiation, if needed [14,15].

Surgical procedure

**Figure 7:**
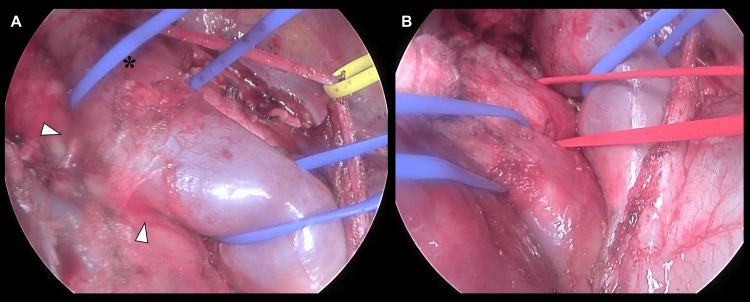
Intraoperative findings (A) Dense adhesions (white arrowheads) are observed on the dorsal aspect of the extrapericardial SVC. The proximal SVC is encircled within the pericardium, and the distal portion is encircled at the level of the brachiocephalic confluence (*). (B) The SVC, right pulmonary veins, and right main pulmonary artery are encircled intrapericardially. SVC: superior vena cava

The patient was positioned in a modified left lateral decubitus position with a 45° posterior pelvic rotation, allowing simultaneous access to the thoracic cavity and femoral vessels (Figure 6A). A 25 cm posterolateral thoracotomy was performed through the fifth intercostal space, extending from the anterior axillary line to 1 cm below the scapular tip, during which an intercostal muscle flap was harvested. Adhesions were observed along the fourth intercostal space of the prior thoracotomy and on the mediastinal side. Particularly dense adhesions were noted around the carina, the SVC, and the hilum, where lymph node dissection had been previously performed. Extensive adhesiolysis allowed the exposure of the SVC, bilateral brachiocephalic veins, and pericardium. Contrary to preoperative expectations, incision of the pericardium posterior to the phrenic nerve revealed no significant intrapericardial adhesions, and the areas surrounding the intrapericardial pulmonary veins and the right main PA were intact. Intrapericardial dissection was therefore initiated without exposure of the femoral vessels for CPB induction. Nevertheless, dense adhesions were encountered involving the extrapericardial SVC, necessitating meticulous vascular control. To ensure safe dissection, the SVC was encircled and controlled both proximally within the pericardium and distally at the level of the brachiocephalic veins (Figure 7A). With proximal and distal control of the SVC established, safe dissection was achieved without hemorrhagic complications. The right upper and lower pulmonary veins were individually secured within the pericardium, and the right main PA could be secured behind the SVC via the pericardium (Figure 7B).

**Figure 8:**
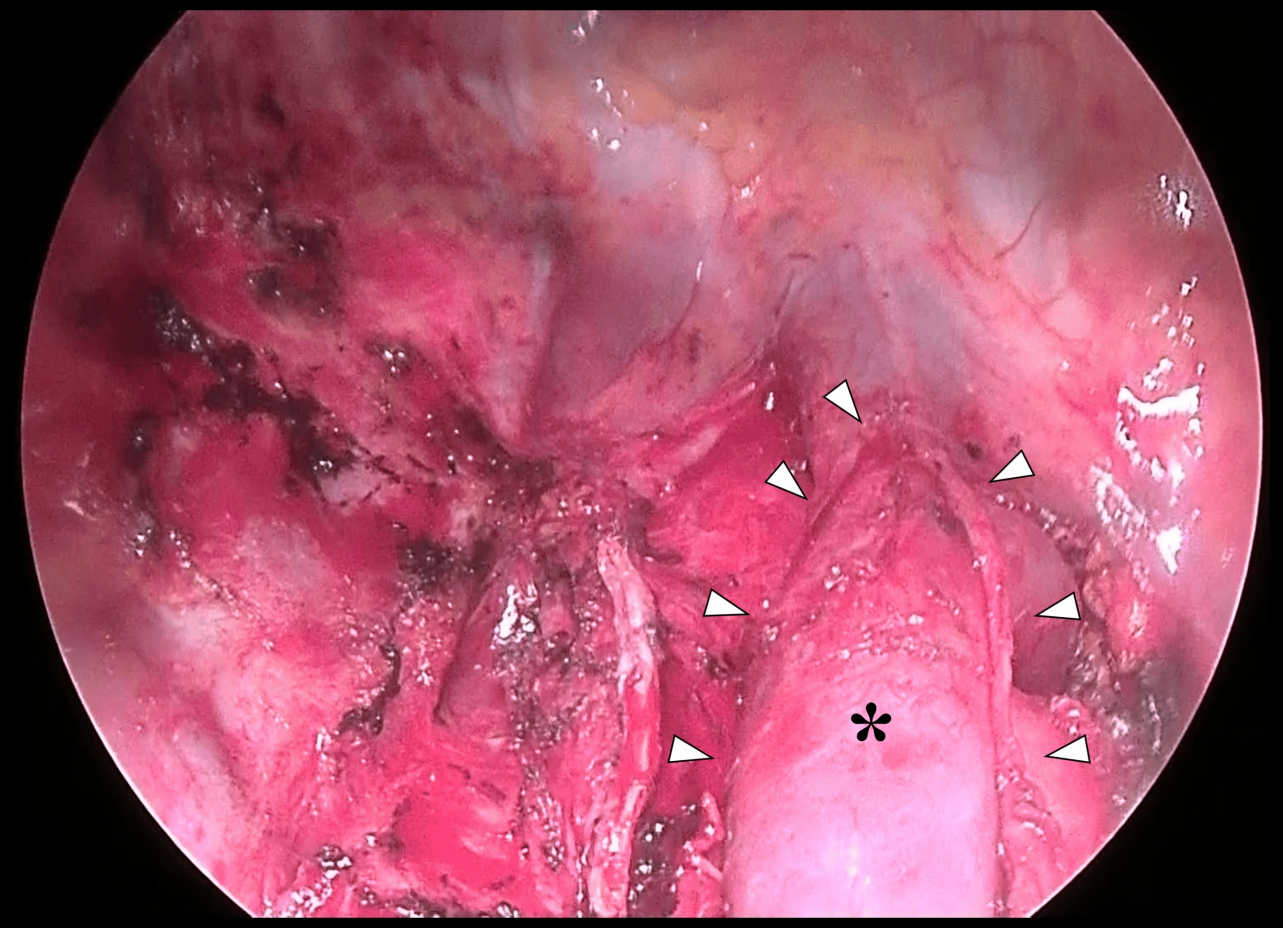
Operative field following right completion pneumonectomy Extensive adhesiolysis is performed and the pericardial cavity is opened. White arrowheads indicate the edges of the incised pericardium, and the asterisk (*) denotes the intrapericardial superior vena cava.

All these vessels were divided using parallel-jaw staplers. The right main bronchus was then divided with a hinged-jaw stapler, allowing the removal of the right lung (Figure 8).

The bronchial stump was reinforced using the harvested intercostal muscle flap and a pericardial fat pad. The pericardial defect was reconstructed with a 0.1 mm expanded polytetrafluoroethylene patch. The total operative time was 329 minutes, the estimated blood loss was 240 mL, and no transfusion was required.

Pathological analysis

**Figure 9:**
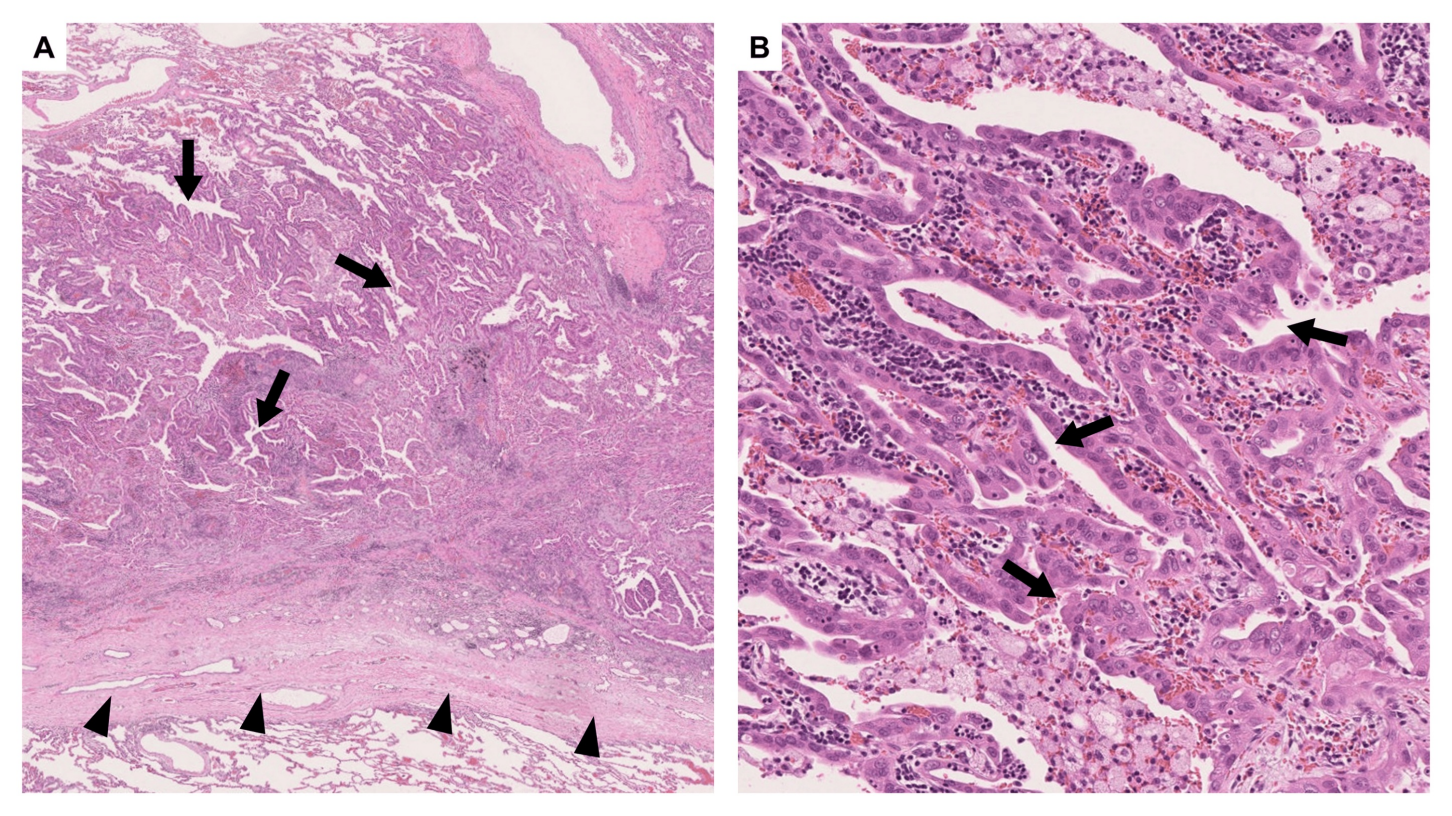
Pathological findings of the right lung The tumor was located along the stapler line (black arrowheads in A) from the previous surgery. The tumor cells exhibited papillary growth pattern (black arrows). (A) Hematoxylin and eosin stain, ×1.25. (B) Hematoxylin and eosin stain, ×10.

Pathological analysis confirmed a 66 mm papillary adenocarcinoma with a 37 mm invasive component. Although lymphovascular invasion was absent, visceral pleural invasion was observed. The tumor extended from the upper to the lower lobe. The histological similarity to the previous middle lobe cancer, combined with its location along the former stapler line, was consistent with local recurrence (Figure 9). Biomarker analysis was not conducted, as no postoperative systemic therapy was planned.

Postoperative course

**Figure 10:**
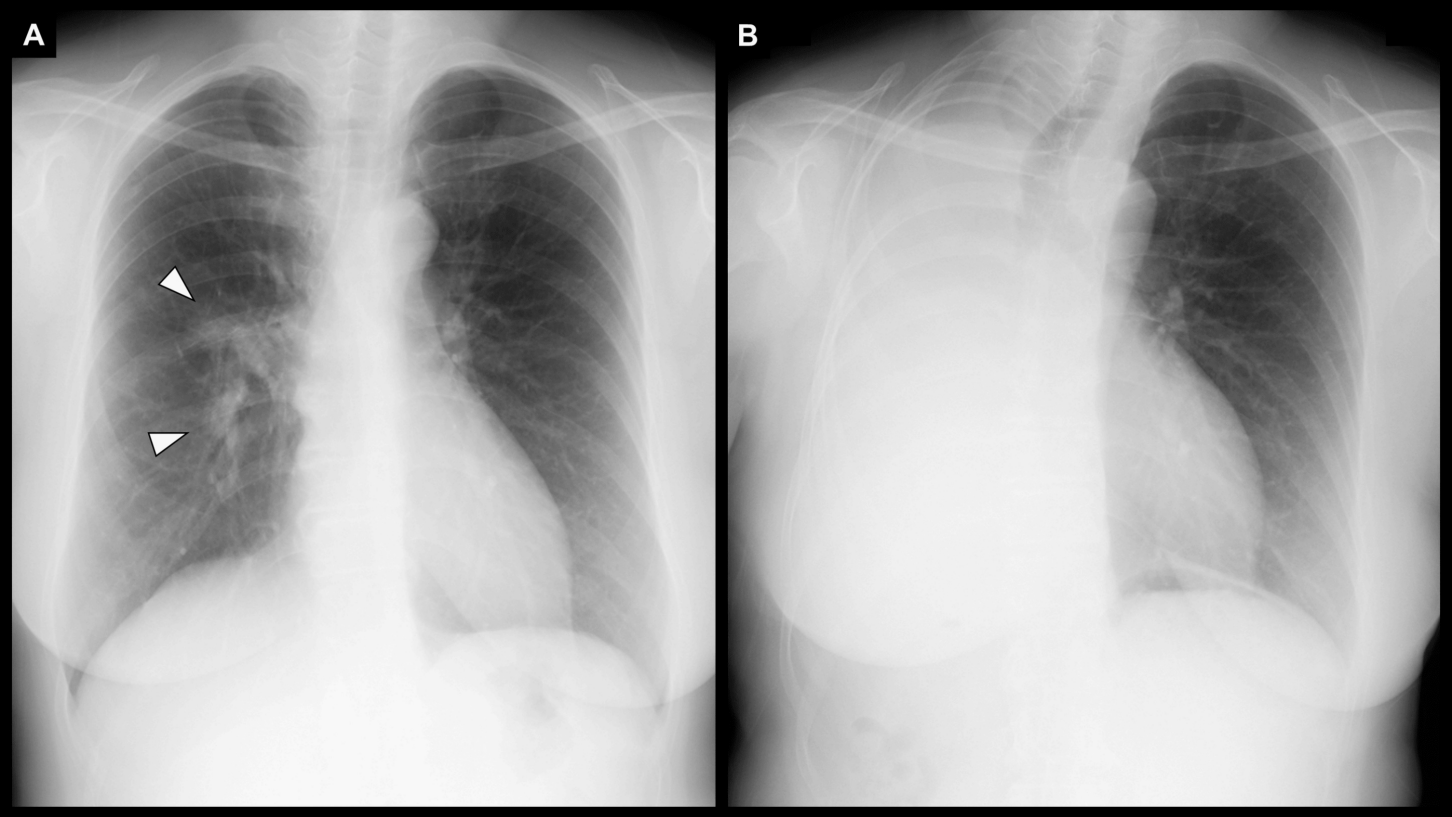
Comparison of chest radiographs before and after right completion pneumonectomy (A) Preoperatively, a mass overlapping the staple line is observed in the right middle lung field (white arrowheads). (B) One year after surgery, the right thoracic cavity is filled with pleural fluid, and a mediastinal shift to the right is observed.

The patient was initially admitted to the intensive care unit and transferred to the general ward on postoperative day (POD) 1 following chest drain removal and her no longer requiring supplemental oxygen. The postoperative course was uneventful, with the patient achieving a stable condition by POD 7. She was observed until POD 15 and subsequently discharged without any acute postoperative complications. Adjuvant chemotherapy was deferred to prioritize the recovery of physical strength following CP. At the one-year follow-up, the patient remained free of long-term complications or recurrence, and she maintained an independent lifestyle and successfully returned to her social activities with an ECOG PS of 0 (Figure 10).

## Discussion

CP is one of the most challenging and hazardous procedures in thoracic surgery, and its success depends on both surgical expertise and comprehensive multidisciplinary planning. As the overall number of pneumonectomies is decreasing due to the rise of parenchymal-sparing techniques, such as sleeve lobectomy, and advancements in multimodality therapies, including neoadjuvant chemotherapy [16], the opportunity to perform CP is becoming even rarer. Despite its clinical importance, standardized protocols for CP are often based on institutional experience, and detailed literature remains scarce. Consequently, CP becomes even more challenging in low-volume centers with limited experience. In this case, a multidisciplinary strategy was implemented preoperatively to prepare for all potential intraoperative contingencies, including critical hemorrhage. We believe it is highly significant that we meticulously planned the operating room layout, cannulation routes including CPB, patient positioning, and the surgical approach while ensuring a shared understanding of the intraoperative decision-making process. The detailed description of these specific preparations provides substantial clinical value. Intrapericardial control of the pulmonary vessels is particularly critical to avoid uncontrollable hemorrhage, especially in patients with intrathoracic dense adhesions due to previous surgeries [3,4]. Provided that the prior surgery has not involved the pericardial cavity, vascular manipulation within the pericardium is not technically demanding. Nevertheless, no preoperative modality can accurately assess this, and the actual status becomes evident only during surgery.

In the present case, a right middle lobectomy and mediastinal lymph node dissection had been performed previously for right middle lobe NSCLC. For middle lobe NSCLC, mediastinal lymph node dissection should be considered a priority in the subcarinal zones (SCZ) than in the upper zones (UZ). Moreover, the hilar zone/interlobar zone is central to unfavorable prognoses in patients with pN2 middle lobe NSCLC [17]. Review of previous operative records revealed that the mediastinal lymph node dissection covered both the UZ and SCZ, extending to the proximal right main PA. This surgical history raised concerns about adhesions between the intrapericardial SVC and the PA. Furthermore, inadvertent opening of the pericardium during mediastinal lymph node dissection is not uncommon; however, it is frequently not explicitly described in operative records. Intrapericardial adhesions induced by these procedures may render vascular manipulation hazardous, necessitating the immediate availability of CPB as a backup.

Percutaneous cardiopulmonary support (PCPS) is also employed in cardiac emergencies. PCPS units are compact, battery-powered, and portable heart-lung machines that can be implemented rapidly in any hospital setting using thin-walled cannulae inserted via the femoral vessels. This system provides temporary circulatory support by actively aspirating blood from the venous system using a centrifugal pump and a hollow-fiber membrane oxygenator for gas exchange [18]. While PCPS is indicated for emergencies such as cardiac arrest and cardiogenic shock, its closed-circuit configuration limits its ability to manage major hemorrhage. In contrast, CPB offers superior capabilities, including active suction, reliable oxygenation, and stable systemic perfusion, making it more suitable for high-risk thoracic procedures in which significant intraoperative bleeding is anticipated [9,10,19-21]. Therefore, CPB was selected as standby support in this case.

To avoid interference of the CPB circuit with the operative field, peripheral cannulation via the femoral artery and vein was planned (Figure 6B). This approach also provided rapid access in the event of an intraoperative emergency [22-24]. The modified lateral decubitus position, inspired by techniques used in thoracoabdominal aortic surgery [25], enabled the simultaneous exposure of both the thorax and femoral vessels. The upper body was positioned in left lateral decubitus with the right arm suspended anteriorly to facilitate posterolateral thoracotomy, while the pelvis was rotated 45° posteriorly to optimize femoral cannulation (Figure 6A). By supporting the body dorsally only at the thoracic and sacral spine, sufficient anterior operative space was ensured without compromising positional stability. In the present case, it was necessary to dissect the proximal right main PA, making the establishment of this ventral surgical field extremely critical.

Central venous catheterization was also strategically planned with particular attention to the risk of SVC injury. Dense extrapericardial SVC adhesions were anticipated due to the prior superior mediastinal lymph node dissection, making further dissection of the SVC potentially hazardous, as bleeding from the SVC in the lateral position could be catastrophic. Therefore, proximal and distal control of the SVC around the adhesion was essential. Consequently, the safest strategy was to secure proximal control within the pericardium and distal control at the level of the brachiocephalic veins. To avoid catheter entrapment during potential SVC clamping, the tip of the central venous catheter was positioned distal to the brachiocephalic confluence.

As total occlusion of the SVC has been reported to be tolerable for up to 30 minutes [26,27], we determined that temporary bypass of the brachiocephalic vein or venous drainage from the internal jugular vein using CPB would not be necessary, even if adhesiolysis required clamping the SVC proximally and distally. Instead, since venous return and intravenous infusion from the upper body would be compromised during SVC occlusion, we secured reliable venous access from the lower body via the femoral vein catheter.

Although CPB was ultimately unnecessary, the comprehensive preoperative planning and interdisciplinary collaboration enabled the safe procedure, resulting in an excellent outcome. Despite its high risk, CP remains necessary in selected clinical scenarios; therefore, preserving the expertise required to perform it safely is essential. This case emphasizes the importance of a preoperative multidisciplinary strategy for meticulous, individualized planning and preparedness for managing the unique technical challenges in CP.

## Conclusions

CP remains a high-risk but indispensable procedure in selected cases. This case underscores the importance of meticulous preoperative planning to achieve safe and successful outcomes. Specifically, surgeons must carefully strategize the potential need for CPB, as well as its implementation regarding cannulation routes, patient positioning, and the choice of surgical approach.
